# Amino Acid Homeostasis and Fatigue in Chronic Hemodialysis Patients

**DOI:** 10.3390/nu14142810

**Published:** 2022-07-08

**Authors:** Adrian Post, Daan Kremer, Dion Groothof, Yvonne van der Veen, Pim de Blaauw, Jennifer van der Krogt, Ido P. Kema, Ralf Westerhuis, M. Rebecca Heiner-Fokkema, Stephan J. L. Bakker, Casper F. M. Franssen

**Affiliations:** 1 Department of Internal Medicine, University of Groningen, University Medical Center Groningen, 9713 GZ Groningen, The Netherlands; d.kremer@umcg.nl (D.K.); d.groothof@umcg.nl (D.G.); y.van.der.veen@umcg.nl (Y.v.d.V.); s.j.l.bakker@umcg.nl (S.J.L.B.); c.f.m.franssen@umcg.nl (C.F.M.F.); 2 Department of Laboratory Medicine, University of Groningen, University Medical Center Groningen, 9713 GZ Groningen, The Netherlands; p.de.blaauw@umcg.nl (P.d.B.); j.van.der.krogt@umcg.nl (J.v.d.K.); i.p.kema@umcg.nl (I.P.K.); m.r.heiner@umcg.nl (M.R.H.-F.); 3 Dialysis Center Groningen, 9713 GZ Groningen, The Netherlands; r.westerhuis@dcg.nl

**Keywords:** amino acids, plasma concentrations, dialysis losses, hemodialysis, fatigue

## Abstract

Patients dependent on chronic hemodialysis treatment are prone to malnutrition, at least in part due to insufficient nutrient intake, metabolic derangements, and chronic inflammation. Losses of amino acids during hemodialysis may be an important additional contributor. In this study, we assessed changes in plasma amino acid concentrations during hemodialysis, quantified intradialytic amino acid losses, and investigated whether plasma amino acid concentrations and amino acid losses by hemodialysis and urinary excretion are associated with fatigue. The study included a total of 59 hemodialysis patients (65 ± 15 years, 63% male) and 33 healthy kidney donors as controls (54 ± 10 years, 45% male). Total plasma essential amino acid concentration before hemodialysis was lower in hemodialysis patients compared with controls (*p* = 0.006), while total non-essential amino acid concentration did not differ. Daily amino acid losses were 4.0 ± 1.3 g/24 h for hemodialysis patients and 0.6 ± 0.3 g/24 h for controls. Expressed as proportion of protein intake, daily amino acid losses of hemodialysis patients were 6.7 ± 2.4% of the total protein intake, compared to 0.7 ± 0.3% for controls (*p* < 0.001). Multivariable regression analyses demonstrated that hemodialysis efficacy (Kt/V) was the primary determinant of amino acid losses (Std. β = 0.51; *p* < 0.001). In logistic regression analyses, higher plasma proline concentrations were associated with higher odds of severe fatigue (OR (95% CI) per SD increment: 3.0 (1.3; 9.3); *p* = 0.03), while higher taurine concentrations were associated with lower odds of severe fatigue (OR (95% CI) per log2 increment: 0.3 (0.1; 0.7); *p* = 0.01). Similarly, higher daily taurine losses were also associated with lower odds of severe fatigue (OR (95% CI) per log2 increment: 0.64 (0.42; 0.93); *p* = 0.03). Lastly, a higher protein intake was associated with lower odds of severe fatigue (OR (95% CI) per SD increment: 0.2 (0.04; 0.5); *p* = 0.007). Future studies are warranted to investigate the mechanisms underlying these associations and investigate the potential of taurine supplementation.

## 1. Introduction

Patients relying on chronic hemodialysis treatment are prone to protein–energy wasting, at least in part due to low nutrient intake, metabolic disturbances, and a chronic inflammatory state [1,2]. Losses of amino acids during hemodialysis may be an important additional contributor. Intradialytic losses of amino acids have been quantified before, ranging from 4 to 13 g per dialysis session [3,4,5,6,7,8,9,10]. It should, however, be noted that most of the aforementioned studies included fewer than ten patients. Similarly, most studies did not include data on urinary losses in patients with residual diuresis. Additionally, it is currently unknown what proportion of the daily intake is lost by dialysis and/or by urinary excretion and what the determinants of these daily losses are.

From a clinical perspective, one of the most debilitating symptoms for hemodialysis patients is fatigue, which affects 42–89% of end-stage kidney disease patients. The relevance of fatigue for patients is emphasized by results from questionnaires which found that 94% of the patients would accept more intense hemodialysis if it would increase their energy level, whereas only 19% would accept this for an increase in longevity by three years [11]. Currently, it is unknown whether alterations in plasma amino acid concentrations or amino acid losses are associated with fatigue.

We aimed to investigate these issues by measuring amino acid concentrations in plasma, urine, and dialysate of hemodialysis patients and amino acid concentrations in plasma and urine in controls, thereby allowing us to compare amino acid plasma concentrations and daily amino acid losses between hemodialysis patients and controls. Furthermore, in the hemodialysis patients, we aimed to determine the intradialytic changes in amino acid plasma concentrations, quantify the total removal of amino acids by dialysis and residual kidney function, and investigate the determinants of the daily amino acid losses. Lastly, we aimed to investigate whether plasma amino acid concentrations, daily amino acid losses, and dietary protein intake are associated with severe fatigue.

## 2. Materials and Methods

### 2.1. Design and Study Population

This observational study was approved by the Medical Ethical Committee of the University Medical Center Groningen, the Netherlands (METc 2015.086, 15 March 2015) and was performed in accordance with standards of ethics laid down in the Declaration of Helsinki in 1964. Each of the participants provided written informed consent. The design of the study has been described previously [12,13,14,15]. Patients undergoing two or three hemodialysis sessions per week were included. For patients dialyzing twice weekly, the last hemodialysis session of the week was used. For patients dialyzing three times per week, the mid-week hemodialysis session was used. Body mass index (BMI) was defined as body weight after dialysis divided by the body height squared.

To compare hemodialysis patients with participants without renal disease, we included 33 healthy kidney donors of whom biomaterial was collected before kidney donation.

### 2.2. Hemodialysis Settings

All studies were performed with the Fresenius 5008 hemodialysis apparatus with a low-flux polysulfone dialyzer (Fresenius Medical Care, Bad Homburg, Germany) using smartbag dialysate concentrations (Fresenius Medical Care, Bad Homburg, Germany), as previously described [12,13,14,15]. Blood flow and dialysate flow were between 200 and 300 mL/min and between 500 and 700 mL/min, respectively. Dialysate temperature was 36.0 or 36.5 °C. Dialysis fluid sodium varied from 136 to 140 mmol/L, potassium from 1 to 3 mmol/L, depending on the patient’s plasma potassium concentration, calcium varied from 1.25 to 1.50 mmol/L, and bicarbonate from 34 to 38 mmol/L.

### 2.3. Sample Collection and Laboratory Measurements

All dialysate was collected in a tank of 200 L. The dialysate was homogenized and sampled for analyses. At the start of hemodialysis and five minutes before the end of the hemodialysis session, blood was drawn from the hemodialysis line. Patients with a residual diuresis of more than 200 mL/24 h were asked to perform two 24-h urine collections before the hemodialysis session of interest.

Plasma and dialysate concentrations of amino acids were measured with a validated hydrophilic interaction liquid chromatography (HILIC) method in combination with tandem mass spectrometry, based on the method of Prinsen et al. [16]. In short, a 20 µL sample was mixed with 20 µL isotopically labeled internal standards and centrifuged. Then, 200 µL methanol was added for protein precipitation, after which samples were centrifuged. One microliter of the supernatant was injected in the UHPLC-MS/MS system. Chromatographic separation was achieved using an ACQUITY UPLC BEH Amide Column (130 Å, 1.7 µm, 2.1 mm × 100 mm) (Waters, Milford, MA, USA) and a Nexera UHPLC system (Shimadzu, Kioto, Japan). The temperature of the analytical column was kept at 40 °C. Mobile phases consisted of 0.1 *v*/*v*% formic acid and 10 mM ammoniumformate in Milli-Q water and 95 *v*/*v*% acetonitrile/Milli-Q water. A Sciex 4500 QTrap mass spectrometer (Sciex, Framingham, MA, USA) was used in positive electrospray ionization mode, using nitrogen as collision, carrier, and curtain gas. Sciex Analyst^®^MD 1.6.2 and Sciex Multiquant^®^MD 3.0 (Sciex, Framingham, MA, USA)were used for data acquisition and processing. Intra-assay and inter-assay coefficients of variation for all the amino acids were <10. Urine amino acid concentrations were measured using a Biochrom amino acid analyzer (Biochrom Ltd., Cambridge, UK). In short, a 50 µL urine sample was injected and the amino acids were separated by ion-exchange chromatography followed by postcolumn derivatization with ninhydrine and spectrophometric detection by 570 and 440 nm, as developed by Moore et al. [17]. A method comparison between the Biochrom and LC-MS/MS method demonstrated adequate comparability. Cysteine was not reported due to instability, aspartic acid was not reported because all concentrations were below the limit of detection. Total tryptophan was measured using a different LC-MS/MS method, as reported previously [14].

Plasma creatinine, urea, and albumin were measured on Roche clinical chemistry analyzers (Modular P/Cobas C, Roche Diagnostics, Mannheim, Germany). Other laboratory measurements were performed with automated and validated routine methods (Roche Diagnostics, Mannheim, Germany). Protein intake was assessed using the urea excretion rate and the Maroni formula [18]: Protein intake = 6.25 × (0.028 × ((V_dialysate_ × Du × n)/7 + UUE) + 0.031 × BW) + UPE, in which V_Dialysate_ = total volume of dialysate (L); Du = measured urea concentration in the collected dialysate (mmol/L); n = number of dialyses per week; UUE = 24 h urine urea excretion (mmol/24 h), averaged from two 24 h urine collections; BW = body weight after hemodialysis (kg); and UPE = 24 h urine protein excretion (g/24 h), averaged from two 24 h urine collections. In controls, the protein intake was calculated using the urinary urea excretion from a single 24 h urine collection and the Maroni formula [18]. The Kt/V was calculated according to the formula of Daugirdas [19]:

Kt/V = −ln (R − 0.008 × t) + (4 − 3.5 × R) × UF/W

in which R is the ratio between the post- and predialysis concentration of urea, t is duration of the hemodialysis session (h), UF is the ultrafiltration volume (L), and W the body weight after hemodialysis (kg).

### 2.4. Comparison of Daily Losses between Hemodialysis Patients and Controls

Due to the fact that hemodialysis treatment was not performed daily and because a proportion of hemodialysis patients had residual diuresis, the intradialytic losses during a single hemodialysis session are not equal to daily amino acid losses. Therefore, we calculated daily amino acid losses using the number of hemodialysis sessions per week and by incorporating urinary amino acid losses. Total daily amino acid losses were calculated in hemodialysis patients as follows:

Daily amino acid losses (μmol/24 h) = ((V_Dialysate_ × (D_x_) × n)/7 + UE_x_)

in which V_Dialysate_ = total volume of the spent dialysate (L); D_x_ = measured concentration of the amino acids in collected dialysate (μmol/L); n = number of dialyses per week; UE_X_ = 24 h urinary amino acid excretion (μmol/24 h), averaged from two 24 h urine collections. In controls, the amino acid concentrations were measured in a single 24 h urine collection and daily losses were calculated as: Daily amino acid losses (μmol/24 h) = V_Urine_ × U_x_, in which V_Urine_ is the urinary volume of the 24 h collection (L) and U_x_ is the amino acid concentration in the urine (μmol/L).

Branched chain amino acids (BCAAs) were calculated as the sum of leucine, isoleucine, and valine. Essential amino acids were calculated as the sum of histidine, leucine, isoleucine, lysine, methionine, phenylalanine, threonine, tryptophan, and valine. Non-essential amino acids were calculated as the sum of alanine, arginine, asparagine, citrulline, glutamic acid, glutamine, glycine, ornithine, proline, serine, taurine, and tyrosine. Total amino acids were calculated as the sum of essential amino acids and non-essential amino acids. 

### 2.5. Intradialytic Changes in Amino Acid Concentrations in Hemodialysis Patients

Intradialytic plasma changes in amino acids were assessed by calculating the absolute change, as well as the proportional change:

Absolute change (μmol/L) = amino acid concentration (μmol/L) after hemodialysis − amino acid concentration (μmol/L) before hemodialysis.

Proportional change (%) = absolute difference (μmol/L)/amino acid concentration (μmol/L) before hemodialysis × 100%.

### 2.6. Single Dialysis Losses, Dialytic Clearance, and Fractional Clearance in Hemodialysis Patients

Single dialysis amino acid losses, expressed as losses per session, are presented separately. The dialytic clearance of individual and summed amino acids was calculated as follows:

Dialytic amino acid clearance (mL/min) = ((V_Dialysate_ × D_x_)/T)/((P_x pre_ + P_x post_)/2) × (1000/60)

In which V_Dialysate_ = total volume of the spent dialysate (L); D_X_ = measured concentration of the amino acids in the collected dialysate (μmol/L); T= the duration of the hemodialysis session (hours); P_x pre_ = plasma concentration the amino acid (µmol/L) before hemodialysis; P_x post_ = plasma concentration of the amino acid (µmol/L) after hemodialysis.

The fractional dialytic clearance of the amino acids was calculated by dividing the dialytic clearance of amino acids by the dialytic creatinine clearance and multiplying by 100%. 

### 2.7. Assessment of Severe Fatigue

The Checklist Individual Strength (CIS) was used to assess the self-reported physical health and mental health. This questionnaire examines fatigue and fatigue-related behavioral aspects using 20 statements which can be answered using a 7-point Likert scale. Higher CIS scores indicate a higher burden of fatigue, and less physical activity, concentration, or motivation, respectively. A subjective fatigue score of ≥35 was defined as severe fatigue [13,20,21]. CIS has been well-validated and is often used in research in patients with various diseases [20,21,22,23,24], including dialysis patients [15,25].

### 2.8. Statistical Analysis

Computations and data analyses were executed using R software (http://cran.r-project.org/ (accessed on 1 May 2022)). Data were presented as mean ± standard deviation for data with a normal distribution, as median [interquartile range] for data not normally distributed, and as numbers (percentages) for nominal data. The distribution of variables was assessed by inspecting histograms and quantile–quantile plots. Difference in baseline characteristics, plasma amino acid concentrations, and daily amino acid losses between hemodialysis patients and controls were tested using independent sample t-tests, the Wilcoxon rank-sum tests, or the chi-squared tests, depending on the type of data and data distribution. Differences in daily amino acid losses, both absolute and relative to the daily protein intake, between hemodialysis patients and controls were graphically demonstrated using a bar plot with error bars. Differences between plasma concentrations before and after hemodialysis were tested using paired sample t-tests or Wilcoxon signed-rank tests, depending on the data distribution.

Linear regression analyses were used to investigate the potential determinants of the total daily amino acid losses in hemodialysis patients. For these analyses, regression coefficients were presented as standardized beta values, referring to the number of standard deviations a dependent variable changes per standard deviation increase in the independent variable, thereby allowing for comparison of the strength of the associations of different potential determinants. Multivariable analyses were performed by including all potential covariates with a *p* < 0.05 in the univariable model. The assumption of linearity was checked visually using scatter plots. The assumption of homoscedasticity was checked using scatter plots of residuals versus predicted values. Non-normally distributed data were log_2_-transformed prior to analysis. Collinearity was checked by calculating the variance inflation factor of each model.

Associations of plasma amino acids, daily amino acid losses, and dietary protein intake with severe fatigue were assessed using logistic regression analyses. The assumption of linearity was checked by visually inspecting scatter plots between the logit of the outcome and the continuous predictor. Collinearity was checked by calculating the variance inflation factor of each model. The logistic regression analyses were performed using two different models with adjustments for a priori defined potential confounders. In model 1, analyses were adjusted for age, sex, BMI, and hemodialysis vintage. In model 2, analyses were adjusted for age, sex, BMI, hemodialysis vintage, hemoglobin concentration, C-reactive protein concentration, cardiovascular disease, and diabetes. To visualize the observed associations with severe fatigue, the relevant continuous variables were individually plotted against the probability of severe fatigue using an inverse logit function and the visreg package in R. These plots demonstrate the effect on the expected value of the response by moving the linear predictor away from a reference point (mean of the variable).

To determine the robustness of the associations found using logistic regression analyses, sensitivity analyses were performed. Sensitivity analyses were performed after (1) excluding outliers of relevant variables, defined as all values deviating more than two standard deviations from the mean, (2) excluding participants with two hemodialysis sessions per week, (3) after excluding participants with a hemodialysis vintage >60 months, (4) excluding participants with a BMI < 18.5 kg/m^2^, and (5) excluding participants with hemoglobin concentrations below the 5th percentile in males and females.

## 3. Results

### 3.1. Baseline Characteristics

A total of 59 hemodialysis patients and 33 controls were included in the study. Compared with controls, hemodialysis patients were on average older and had a lower urinary volume (Table 1, all *p* < 0.05). Hemodialysis patients and controls were of similar weight and height and had similar BMI (all *p* > 0.05). Median hemodialysis vintage was 15 (6 – 39) months and nearly all (95%) hemodialysis patients dialyzed thrice weekly and most patients (81%) dialyzed for four hours per session. Mean Kt/V was 1.4 ± 0.3 per session with an average ultrafiltration volume of 1.9 ± 0.9 L. A total of 32 (54%) patients had residual diuresis, with a mean urinary volume of 0.9 ± 0.6 L. 

Protein intake was significantly lower in the hemodialysis patients compared with the controls, both absolute (64 ± 21 vs. 84 ± 21 g/24 h; *p* < 0.001) and expressed per kg bodyweight (0.82 ± 0.23 vs. 1.11 ± 0.21 g/kg/24 h; *p* < 0.001).

### 3.2. Comparison of Amino Acid Concentrations between Hemodialysis Patients and Controls

A comparison of plasma amino acid concentrations between hemodialysis patients before hemodialysis and controls is shown in Table 2. Plasma concentrations before hemodialysis of lysine, methionine, threonine, tryptophan, valine, alanine, asparagine, glutamine, serine, and tyrosine were all lower in hemodialysis patients compared to controls (all *p* < 0.05), whereas plasma concentrations of phenylalanine, citrulline, glutamic acid, glycine, and taurine were higher in hemodialysis patients when compared to controls (all *p* < 0.05). Analysis of summed concentrations demonstrated that the concentrations of total essential amino acids were lower in hemodialysis patients as compared to controls (*p* = 0.006). No differences between hemodialysis patients and healthy controls were found for the summed BCAAs, non-essential amino acids, and total amino acid concentrations (*p* > 0.05).

### 3.3. Daily Amino Acid Losses in Hemodialysis Patients and Controls

**Figure 1:**
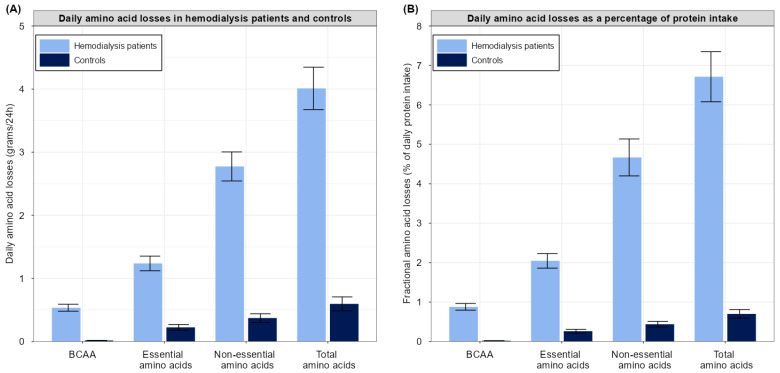
Comparison of absolute daily amino acid losses (**A**) and daily amino acid losses expressed as a percentage of daily protein intake (**B**) in hemodialysis patients and controls. Error bars refer to lower and upper Gaussian confidence limits based on the t-distribution. Abbreviations: BCAA: Branched chain amino acid.

The comparison of daily amino acid losses between hemodialysis patients (sum of dialytic and urinary losses) and controls (only urinary losses) is shown in Table 3. Daily amino acid losses were all higher in hemodialysis patients than in controls (all *p* < 0.001; except loss of histidine which was only numerically higher, *p* = 0.07). An overview of summed daily amino acid losses expressed in grams, as well as expressed as a percentage of daily protein intake, is shown in Table S1. Total daily amino acid losses were 4.0 ± 1.3 g in hemodialysis patients versus 0.6 ± 0.3 g of amino acids in controls (*p* < 0.001), corresponding to a 7-fold higher daily amino acid loss in hemodialysis patients. Expressed as a percentage of daily protein intake, hemodialysis patients lost 6.7 ± 2.4% amino acids per day, while controls lost 0.7 ± 0.3% amino acids per day (*p* < 0.001), corresponding to a 10-fold higher percentual loss in hemodialysis patients. A graphical representation of the comparison of amino acid losses between hemodialysis patients and controls is shown in Figure 1.

### 3.4. Determinants of Total Amino Acid Losses in Hemodialysis Patients

Analyses of potential determinants of the total amino acid losses (in g/24 h) in hemodialysis patients are shown in Table S2. Univariably, total daily amino acid losses were associated with age (Std. β: −0.27; *p* = 0.04), Kt/V (Std. β: −0.46; *p* = 0.001), plasma albumin (Std. β: 0.31; *p* = 0.02), total creatinine excretion (Std. β: 0.35; *p* = 0.006), and protein intake (Std. β: 0.32; *p* = 0.02). Multivariable linear regression analyses, including each of the aforementioned variables, demonstrated that total daily amino acid losses were significantly associated with Kt/V (Std. β: 0.51; *p* < 0.001), but no longer associated with age, plasma albumin, total creatinine excretion, or protein intake (all *p* > 0.05).

### 3.5. Intradialytic Changes in Plasma Concentrations in Hemodialysis Patients

A comparison of amino acid plasma concentrations before and after hemodialysis is shown in Table 4. During hemodialysis, plasma concentrations of histidine, threonine, valine, alanine, arginine, citrulline, glutamine, glycine, ornithine, proline, and taurine significantly decreased (all *p* < 0.05), whereas plasma concentrations of isoleucine, leucine, and tryptophan significantly increased (all *p* < 0.05). Summed concentrations demonstrated that concentrations of non-essential amino acids and total amino acids decreased during hemodialysis (both *p* < 0.05), while levels of BCAAs and essential amino acids did not significantly change (both *p* > 0.05). 

### 3.6. Single Hemodialysis Amino Acid Losses, Dialytic Amino Acid Clearances, and Relative Contribution of Dialysis and Urine to Daily Losses

Individual amino acid losses during a single hemodialysis session, the clearance of amino acids by hemodialysis, and the fractional amino acid clearance (relative to creatinine clearance) are shown in Table S3.

During a single hemodialysis session, BCAA losses were 1.3 ± 0.5 g, essential amino acid losses were 2.9 ± 1.0 g, non-essential amino acid losses were 6.4 ± 2.0 g, and total amino acid losses were 9.3 ± 2.9 g.

The dialytic amino acid clearances were mostly similar to the clearance of creatinine (137 ± 68 mL/min), however, lower clearances were found for tryptophan (80 ± 21 mL/min), glutamic acid (81 ± 35 mL/min), and taurine (20 (8; 51) ml/min). In a total of 32 hemodialysis patients with residual diuresis, a comparison between dialysis and urinary losses of amino acids is demonstrated in Table S4. The median ratios between hemodialysis losses to urinary losses were 132, 27, 16, and 17 for BCAAs, essential amino acids, non-essential amino acids, and total amino acids, respectively.

### 3.7. Associations of Plasma Amino Acid Concentrations with Severe Fatigue

Logistic regression analyses on the associations of plasma amino acid concentrations with severe fatigue are demonstrated in Table 5. A total of 20 (38%) hemodialysis patients suffered from severe fatigue. Higher plasma proline concentrations were associated with higher odds of severe fatigue (model 1 OR (95%CI) per 1 SD increment: 2.6 (1.2; 7.1); *p* = 0.04). After further adjustment for potential confounders, including, amongst others, hemoglobin concentration, the association remained materially unchanged (model 2 OR: 3.0 (1.3; 9.3); *p* = 0.03). The association lost statistical significance in sensitivity analysis where outliers were excluded, in sensitivity analysis where patients with two hemodialysis sessions per week were excluded, and in sensitivity analysis where participants with a hemodialysis vintage >60 months were excluded. 

**Figure 2:**
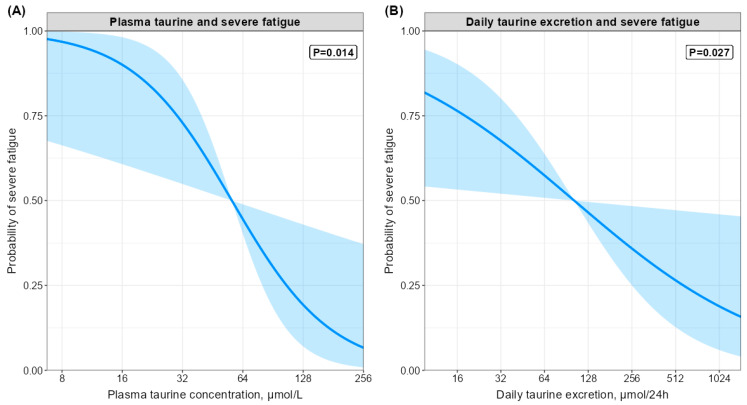
Graphical representation of the association of plasma taurine (**A**) and daily taurine excretion (**B**) with the probability of severe fatigue. The analyses are adjusted for age, sex, body mass index, dialysis vintage, hemoglobin concentration, C-reactive protein concentration, presence of cardiovascular disease, and presence of diabetes.

Higher plasma taurine concentrations were associated with lower odds of severe fatigue (model 1 OR (95%CI) per log_2_ increment: 0.4) (0.2; 0.8); *p* = 0.03), independent of age, sex, body mass index, and dialysis vintage. After further adjustment for potential confounders, including hemoglobin concentration, C-reactive protein, cardiovascular disease, and diabetes, the association remained materially unchanged (model 2 OR (95% CI): 0.3 (0.1; 0.7); *p* = 0.01). Plasma concentrations of none of the other individual amino acids, nor summed amino acids, were associated with severe fatigue (all *p* > 0.05). In a sensitivity analysis where outliers were removed, the association of proline with severe fatigue lost significance, while the association of plasma taurine concentration with severe fatigue remained significant throughout all sensitivity analyses (Table S5). The association of plasma taurine concentration with severe fatigue also remained unchanged after adjusting for dietary protein intake (data not shown). A graphical representation of the association between plasma taurine concentration and severe fatigue is shown in Figure 2A.

### 3.8. Associations of Daily Amino Acids Losses and Protein Intake with Severe Fatigue

Logistic regression analyses on the associations of daily amino acid losses and protein intake with severe fatigue are demonstrated in Table 6. Higher daily taurine losses were associated with lower odds of severe fatigue (model 1 OR (95%CI) per log_2_ increment: 0.7 (0.5; 1.0); *p* = 0.04), independent of age, sex, body mass index, and dialysis vintage. After further adjustment for potential confounders, including hemoglobin concentration, C-reactive protein, cardiovascular disease, and diabetes, the association remained materially unchanged (model 2 OR (95% CI): 0.6 (0.4; 0.9); *p* = 0.03). A graphical representation of the association between daily taurine losses and severe fatigue is shown in Figure 2B. None of the other individual amino acid losses or summed amino acid losses were associated with severe fatigue (all *p* > 0.05).

Higher protein intake was associated with lower odds of severe fatigue (model 1 OR (95% CI) per 1 SD increment: 0.2 (0.04; 0.5); *p* = 0.003). After further adjustment for potential confounders, the association remained materially unchanged (OR (95% CI): 0.2 (0.04; 0.5); *p* = 0.007). Lastly, a higher protein intake indexed to bodyweight was also associated with lower odds of severe fatigue, independent of potential confounders, including, amongst others, hemoglobin concentration. The associations of higher protein intake, both absolute and indexed to bodyweight, remained significant in all sensitivity analyses (Table S5). A graphical representation of the association of protein intake and protein intake indexed to bodyweight with severe fatigue is shown in Figure S1.

## 4. Discussion

By measuring amino acid concentrations in plasma, urine, and dialysate of hemodialysis patients and controls, we were able to perform a comprehensive comparison of amino acid homeostasis in hemodialysis patients and controls. We demonstrated that plasma concentrations of essential amino acids are lower in hemodialysis patients as compared to controls, while no differences were found for non-essential amino acids. During hemodialysis, most amino acid concentrations decreased, whereas the concentrations of isoleucine, leucine, and tryptophan increased. During a single hemodialysis session, hemodialysis patients lost 9.3 g of amino acids. Taking into account the number of hemodialysis sessions per week and the urinary losses, the total daily amino acid losses of hemodialysis patients were 4.0 ± 1.3 g/24 h, which is seven times higher than the loss of 0.6 ± 0.3 g/24 h observed in controls. By expressing total daily amino acids losses as a portion of the protein intake, daily amino acid losses were 6.7%, compared to 0.7% in controls. While controlling for the effect of important confounders, higher dialysis efficacy (Kt/V) was associated with larger daily amino acid losses. Higher plasma taurine concentrations and higher daily taurine losses were both associated with lower odds of severe fatigue, independent of confounders. Furthermore, a higher protein intake was strongly associated with reduced odds of severe fatigue.

Under physiological circumstances, the kidneys are not only important for filtration and reabsorption of amino acids, but also play an important role in the synthesis and interorgan exchange of several amino acids. Comparison of plasma amino acid concentrations between hemodialysis patients and controls demonstrated lower plasma concentrations of essential amino acids in hemodialysis patients. Furthermore, compared with controls, hemodialysis patients had 41% lower serine concentrations, whereas glycine concentrations were 21% higher. Physiologically, the kidney is a major source for the endogenous production of serine from glycine. This production is mediated by the glycine cleavage enzyme complex and serine hydroxymethyltransferase in the proximal tubules [26,27]. Impaired kidney function comprises both a decline in glomerular filtration rate as well as loss of functional enzymatic capacity of the kidney. It is therefore likely that reduced functioning kidney mass in hemodialysis patients explains the lower serine and higher glycine concentrations, as has previously been reported [28]. A comparable phenomenon may explain the high citrulline concentration in hemodialysis patients compared to controls. Under normal circumstances, glutamine in the gut is metabolized to citrulline and ammonia and subsequently, citrulline passes the liver without uptake and is taken up by the kidneys where it is converted to arginine, making kidneys the main site for de novo arginine synthesis [27,29]. Impaired kidney-dependent conversion of citrulline to arginine may explain the higher citrulline concentration in hemodialysis patients as compared to controls. Likewise, for a long time it was assumed that enzymatic conversion of phenylalanine to tyrosine by phenylalanine 4-hydroxylase was exclusively a function of the liver. However, previous research has shown phenylalanine 4-hydroxylase activity in the kidney [30]. The relevance of this finding was confirmed by showing in vivo renal conversion of phenylalanine to tyrosine using stable isotope techniques [31]. This may therefore explain our finding of higher phenylalanine and lower tyrosine plasma concentrations in hemodialysis patients, as compared to controls.

Whereas most amino acids demonstrated a decrease during hemodialysis, leucine, isoleucine, and tryptophan demonstrated a significant increase during hemodialysis. While we are unable to explore the underlying mechanisms, there are hypotheses for these changes. Tryptophan is heavily protein bound and before hemodialysis there is competition of uremic toxins and tryptophan for binding spots on plasma proteins such as albumin [32]. During hemodialysis, removal of uremic toxins frees up binding spots and may lead to an extravascular to intravascular shift of tryptophan. Furthermore, ultrafiltration leads to hemoconcentration, increasing the concentration of plasma proteins. Combined, these factors may lead to an increase in total tryptophan concentration during hemodialysis. Another potential mechanism may be muscle mass breakdown of amino acids during hemodialysis, although this does not explain the differential change in amino acids.

Under physiologic circumstances, the vast majority of amino acids are reabsorbed in the proximal tubules after glomerular filtration, causing only minimal amounts of amino acids to be excreted into urine [33]. Previous studies on urinary amino acid losses in healthy individuals demonstrated that the urinary losses account for roughly 0.5% of total dietary protein intake [34], which is in line with our findings in controls of 0.7% of total dietary protein intake in controls. In contrast, for hemodialysis patients the total daily amino acid losses accounted for 6.7% of the total dietary protein intake, the majority of which was lost via hemodialysis. It should be noted that the actual losses might be higher, as our analyses did not measure small proteins and peptides lost during the hemodialysis session. The seven times higher losses of amino acids underscore the need for higher protein intakes in hemodialysis patients. Besides the higher losses of amino acids compared to controls, hemodialysis patients are also in need of more protein intake due to the catabolic (or antianabolic) state induced by the uremic milieu, the oxidative and carbonyl stress, the inflammation, as well as the bioincompatible materials patients are exposed to during the hemodialysis sessions [35]. Current recommendations for dietary protein intake are based on a one size fits all principle and recommend a protein intake of 1.2 g/kg bodyweight per day [36], which are often not met, including in our study where the vast majority did not reach 1.2 g/kg/24 h. The impact of this lower protein intake is highlighted by the logistic regression analyses demonstrating that higher protein intake was strongly associated with lower odds of severe fatigue.

Fatigue is an (often) under-recognized and under-treated symptom, yet one of the most debilitating symptoms for hemodialysis patients. A possible mechanism of the association between protein intake and severe fatigue may be preservation of muscle mass and muscle strength, contributing to better physical functioning and, as a consequence, less fatigue. Other possibilities are that higher protein intake may prevent or counteract protein–energy wasting, which is also associated with fatigue, or that the pathophysiologic causes of fatigue, such as chronic inflammation, can lead to both fatigue and a poor appetite. An association between protein intake and fatigue has also been reported previously in kidney transplant recipients [37]. Based on our findings that Kt/V is the primary determinant for amino acid losses in hemodialysis patients, and in line with a trend towards more personalized medicine, it may be beneficial to adapt the recommended protein intake based on the current Kt/V value. Further studies are warranted to determine what an optimal protein intake per Kt/V increase would be and whether this strategy is associated with improved physical functioning.

Due to the relatively large losses of amino acids, we expected that lower plasma amino acid concentrations would also be associated with higher odds of fatigue. Instead, an association of higher plasma proline with higher odds of severe fatigue was found. While no previous studies have investigated this association before in hemodialysis patients, a study in 160 elderly patients found that higher plasma proline concentrations were associated with higher odds of sarcopenia [38]. The study performed a comprehensive geriatric assessment, which included an evaluation of hand grip strength, gait speed, and muscle mass. The plasma concentrations of 18 amino acids were measured. Multivariable analysis revealed that only a higher concentration of proline was independently associated with sarcopenia [38]. Another study demonstrated that lower plasma proline concentrations were associated with higher physical activity and shorter sitting times in patients with chronic obstructive pulmonary disease [39]. Lastly, significantly higher proline concentrations were observed in COPD patients with cachexia [40]. Proline is a glycogenic amino acid, and may provide a possible link between dysregulated skeletal muscle metabolism and glucose homeostasis in situations of protein–energy wasting. However, the underlying mechanisms of the found associations remain unknown.

Taurine is an amino sulfonic acid found in high concentrations in many cells, where it is involved in a plethora of physiological functions. In certain species, such as cats, taurine is an essential nutrient, but in humans, it is considered a conditionally essential nutrient [41,42,43]. Taurine can be taken up from the diet or synthesized endogenously. Due to low activity of the rate limiting step in the conversion of cysteine to taurine, cysteine sulfinic acid decarboxylase, endogenous synthesis is limited in humans [41,44,45]. Therefore, daily taurine losses mostly reflect dietary taurine intake, with important sources being seafood, poultry, beef, pork, processed meats, energy drinks, and, to a lesser degree, dairy [44]. The 60% lower daily taurine losses in hemodialysis patients therefore likely indicate that taurine consumption is lower in hemodialysis patients than controls. In contrast, plasma taurine concentrations were higher in hemodialysis patients than controls, which is in contrast to a previous study [46], but in line with another [47]. In our study, both higher plasma taurine concentration and higher daily taurine losses were associated with lower odds of severe fatigue. Although it would theoretically be reasonable to expect that higher dietary taurine intake would lead to higher plasma taurine concentration, we found no significant correlation between plasma taurine concentration and daily taurine losses, suggesting that they reflect different processes.

There have been various studies investigating the potential of taurine in improving performance of athletes and young individuals, which has extensively been reviewed by Kurtz et al. [48]. In short, taurine supplementation demonstrated improvements in VO_2_max and time to exhaustion [49,50], 3 or 4 km time-trial [51,52], anaerobic performance [50,53], peak power [50,54], and recovery [55]. Approximately two thirds of the studies reviewed by Kurt et al. reported improvements to performance variables [48]. However, the study designs, subject population, and experimental outcomes were too variable to draw conclusions. Potential mechanisms for effects of taurine include beneficial effects on glucose and lipid regulation, energy metabolism, anti-inflammatory modulation, modulation of ion flux, Ca^2+^ homeostasis, membrane stabilization, and antioxidant actions [48,56,57,58]. While we were unable to investigate the mechanisms underlying the associations with fatigue, the combination of both higher plasma taurine concentration and higher daily taurine losses being associated with severe fatigue warrants further investigation into the potential of taurine supplementation in hemodialysis patients.

Strengths of the study are the assessment of amino acids in plasma, urine, and dialysate, allowing for a comprehensive analysis, and the use of a biomarker-based method to assess dietary protein intake, thereby avoiding potential biases of classic dietary assessments. In addition, we collected the total dialysate instead of taking several samples during the hemodialysis sessions, thereby increasing the accuracy of our amino acid losses quantifications. However, we acknowledge that our study has limitations. While we adjusted for potential confounders, including dialysis vintage, hemoglobin concentration, and relevant comorbidities, we lacked data on psychological factors that may contribute to fatigue [59]. Our study included hemodialysis with two and three hemodialysis sessions per week, reflecting the overall population in clinical practice. Hemodialysis patients with only two hemodialysis sessions per week are often in better condition, which may influence the results. However, in our sensitivity analyses we demonstrated that the associations of plasma taurine concentration, daily taurine losses, and protein intake with severe fatigue remained significant after excluding participants with two hemodialysis sessions per week. Unfortunately, we were unable to report data on cysteine and aspartic acid. Cysteine is well-known for its instability, and many subjects had aspartic acid values below the detection limit, thereby precluding us from depicting these data. Furthermore, due to the observational nature, we were unable to determine the mechanisms of the found associations. Lastly, statistical significance does not per se translate into biological relevance. Nonetheless, the found associations of plasma proline and taurine are plausible based on previous research [38,39,40,49,50,51,52].

In conclusion, we demonstrated that plasma concentrations of total essential amino acids are lower in hemodialysis patients as compared to controls, while no differences were found for total non-essential amino acids. On a daily basis, total amino acid losses in hemodialysis patients account for 6.7% of the total protein intake, whereas in controls they are merely 0.7%. Determinant analyses demonstrated that a higher dialysis efficacy (Kt/V) is the primary determinant of amino acid losses. Higher protein intake was strongly associated with reduced odds of severe fatigue. Plasma concentrations of most amino acids were not associated with the odds of severe fatigue. Interestingly, both plasma concentrations and total taurine losses were associated with severe fatigue, independent of age, sex, body mass index, dialysis vintage, hemoglobin concentration, C-reactive protein concentration, presence of cardiovascular disease, and presence of diabetes. These findings highlight the potential clinical relevance of adequate protein intake and taurine plasma concentration and intake in hemodialysis patients. Future studies are warranted to investigate the mechanisms underlying the observed associations. 

## Figures and Tables

**Table 1:** Baseline characteristics of the hemodialysis patients and controls.

	Hemodialysis Patients (*n* = 59)	Controls (*n* = 33)	*p*-Value
**Demographics**			
Age, years	65 ± 15	54 ± 10	<0.001
Sex, *n* male (%)	37 (63)	15 (45)	0.2
**Dialysis-related**			
Dialysis vintage, months	15 (6; 39)	–	–
Dialysis sessions, *n* (%)			
Two sessions per week	3 (5)	–	–
Three sessions per week	56 (95)	–	–
Dialysate volume, L	135 ± 27	–	–
Ultrafiltration volume, L	1.9 ± 0.9	–	–
Kt/V	1.4 ± 0.3	–	–
Residual diuresis, *n* (%)	32 (54)	33 (100)	<0.001
Urinary volume ^1^, L	0.9 ± 0.6	2.5 ± 0.9	<0.001
Urinary protein excretion, g/24 h	0.7 (0.3; 1.3)		
Plasma albumin, g/L	39 ± 5	45 ± 2	<0.001
Hemoglobin, mmol/L	6.9 ± 0.7	8.8 ± 0.8	<0.001
**Body composition**			
Weight predialysis ^2^, kg	80 ± 16	77 ± 17	0.3
Weight postdialysis, kg	78 ± 16	–	–
Height, m	1.75 ± 0.09	1.72 ± 0.10	0.2
BMI ^3^, kg/m^2^	25.5 ± 4.3	25.6 ± 4.3	0.4
**Protein intake**			
Protein intake absolute, g/24 h	64 ± 21	84 ± 21	<0.001
Protein intake per kg bodyweight, g/kg/24 h	0.82 ± 0.23	1.11 ± 0.21	<0.001

^1^ Refers to hemodialysis patients with residual diuresis. ^2^ For controls this refers to body weight. ^3^ In hemodialysis patients calculated using postdialysis bodyweight. Differences are tested using independent sample t-test, Wilcoxon rank-sum test, or chi-squared test. Abbreviations: BMI: Body mass index.

**Table 2:** Comparison of plasma amino acid concentrations between hemodialysis patients and controls.

Metabolite	Predialysis Concentration in Hemodialysis Patients (μmol/L)	Concentration in Healthy Controls (μmol/L)	Proportional Difference *	*p*-Value
**Essential**				
Histidine	69 ± 19	74 ± 12	–7%	0.2
Isoleucine	63 ± 23	61 ± 18	+3%	0.6
Leucine	103 ± 41	110 ± 29	–6%	0.4
Lysine	141 ± 36	171 ± 37	–18%	<0.001
Methionine	19 ± 7	21 ± 5	–10%	0.03
Phenylalanine	68 ± 22	57 ± 9	+19%	0.002
Threonine	87 ± 29	115 ± 24	–24%	<0.001
Tryptophan	22 (20; 28)	54 (46; 57)	–59%	<0.001
Valine	186 ± 59	213 ± 51	–13%	0.03
**Non-essential**				
Alanine	351 ± 135	423 ± 113	–17%	0.01
Arginine	77 ± 22	78 ± 17	–1%	0.7
Asparagine	53 ± 16	61 ± 11	–13%	0.004
Citrulline	78 (61; 95)	28 (24; 32)	+179%	<0.001
Glutamic acid	101 (69; 161)	57 (45; 81)	+78%	<0.001
Glutamine	514 ± 111	575 ± 87	–11%	0.008
Glycine	260 ± 84	202 ± 54	+29%	<0.001
Ornithine	63 ± 20	59 ± 16	+7%	0.4
Proline	276 (223; 322)	248 (197; 321)	+11%	0.3
Serine	61 ± 19	103 ± 19	–41%	<0.001
Taurine	58 (41; 87)	40 (37; 50)	+45%	0.014
Tyrosine	45 ± 16	62 ± 18	–27%	<0.001
**Total amino acids**				
Total BCAAs	353 ± 119	384 ± 94	–9%	0.2
Total essential	761 ± 200	875 ± 155	–13%	0.006
Total non-essential	2052 ± 587	1977 ± 328	+4%	0.4
Total all amino acids	2813 ± 693	2852 ± 427	–1%	0.7

Differences are tested using independent sample t-test or Mann–Whitney U test. * Proportional difference is defined as: Summary statistic concentration in hemodialysis patients minus concentration in controls divided by the concentration in controls. Data are presented as mean ± standard deviation for data with a normal distribution and as median (interquartile range) for data not normally distributed. Abbreviations: BCAAs: Branched chain amino acids.

**Table 3:** Comparison of daily amino acid losses between hemodialysis patients and controls.

Metabolite	Daily Losses in Hemodialysis Patients (μmol/24 h)	Daily Losses in Controls (μmol/24 h)	Ratio of Hemodialysis to Controls	*p*-Value
**Essential**				
Histidine	862 ± 270	698 ± 463	1.2	0.07
Isoleucine	799 ± 338	12 ± 7	67	<0.001
Leucine	1382 ± 587	67 ± 23	21	<0.001
Lysine	1505 (1233; 1845)	209 (157; 288)	7	<0.001
Methionine	172 ± 95	5 ± 2	34	<0.001
Phenylalanine	729 (631; 967)	68 (52; 103)	11	<0.001
Threonine	1043 (731; 1279)	105 (81; 161)	10	<0.001
Tryptophan	216 ± 63	148 ± 57	1.5	<0.001
Valine	2017 (1606; 2614)	42 (35; 54)	45	<0.001
**Non-essential**				
Alanine	3926 ± 1548	361 ± 219	11	<0.001
Arginine	826 ± 377	34 ± 15	24	<0.001
Asparagine	747 ± 290	122 ± 82	6	<0.001
Citrulline	463 (347; 570)	11 (7; 13)	42	<0.001
Glutamic acid	966 ± 559	35 ± 33	28	<0.001
Glutamine	6954 ± 2410	449 ± 211	15	<0.001
Glycine	3502 ± 1271	1521 ± 848	2	<0.001
Ornithine	541 ± 194	22 ± 17	25	<0.001
Proline	3601 ± 1351	8 ± 5	450	<0.001
Serine	754 (572; 1004)	324 (236; 467)	2	<0.001
Taurine	132 (24; 235)	339 (204; 668)	0.4	<0.001
Tyrosine	462 ± 192	105 ± 68	4	<0.001
**Totals**				
Total BCAAs	4307 ± 1717	125 ± 52	34	<0.001
Total essential	9060 ± 3287	1463 ± 862	6	<0.001
Total non-essential	23,059 ± 7284	3837 ± 1939	6	<0.001
Total losses	32,119 ± 10,221	5300 ± 2749	6	<0.001

Differences are tested using independent sample t-test or Mann–Whitney U test. Data are presented as mean ± standard deviation for data with a normal distribution and as median (interquartile range) for data not normally distributed. Abbreviations: BCAAs: Branched chain amino acids.

**Table 4:** Overview of intradialytic changes in plasma amino acid concentrations in hemodialysis patients.

Metabolite	Predialysis Concentration (μmol/L)	Postdialysis Concentration (μmol/L)	Absolute Change (μmol/L)	Proportional Difference	*p*-Value
**Essential**					
Histidine	69 ± 19	54 ± 14	–15 ± 13	–22%	<0.001
Isoleucine	63 ± 23	74 ± 22	+11 ± 26	+17%	0.002
Leucine	103 ± 41	123 ± 38	+20 ± 46	+20%	0.001
Lysine	141 ± 36	133 ± 37	–7 ± 38	–5%	0.14
Methionine	19 ± 7	19 ± 6	+1 ± 7	+5%	0.46
Phenylalanine	68 ± 22	65 ± 13	–3 ± 18	–4%	0.27
Threonine	87 ± 29	80 ± 22	–7 ± 22	–8%	0.02
Tryptophan	22 (20; 28)	27 (24; 34)	+5 (0; 15)	+23%	<0.001
Valine	186 ± 59	146 ± 37	–38 ± 52	–20%	<0.001
**Non-essential**					
Alanine	351 ± 135	251 ± 85	–99 ± 110	–28%	<0.001
Arginine	77 ± 22	60 ± 19	–17 ± 23	–22%	<0.001
Asparagine	53 ± 16	50 ± 15	–3 ± 14	–6%	0.10
Citrulline	78 (61; 95)	34 (27; 46)	–42 (27; 50)	–54%	<0.001
Glutamic acid	101 (69; 161)	108 (64; 138)	+2 (–26; 14)	+2%	0.78
Glutamine	514 ± 111	457 ± 96	–58 ± 84	–11%	<0.001
Glycine	260 ± 84	203 ± 55	–57 ± 57	–22%	<0.001
Ornithine	63 ± 20	51 ± 12	–12 ± 17	–19%	<0.001
Proline	276 (223; 322)	230 (212; 280)	–33 (– 83; 16)	–12%	0.002
Serine	61 ± 19	60 ± 18	–1 ± 13	–2%	0.54
Taurine	58 (41 − 87)	35 (23; 53)	–17 (–4; –39)	–29%	<0.001
Tyrosine	45 ± 16	45 ± 12	–0 ± 16	–0%	0.97
**Total amino acids**					
Total BCAAs	353 ± 119	343 ± 94	–7 ± 119	–3%	0.7
Total essential	761 ± 200	724 ± 170	–34 ± 206	–5%	0.2
Total non-essential	2052 ± 587	1664 ± 309	–384 ± 510	–19%	<0.001
Total amino acids	2813 ± 693	2388 ± 442	–418 ± 637	–15%	<0.001

Differences are tested using paired sample t-test or Wilcoxon signed-rank test. Data are presented as mean ± standard deviation for data with a normal distribution and as median (interquartile range) for data not normally distributed. Abbreviations: BCAAs: Branched chain amino acids.

**Table 5:** Logistic regression analyses of plasma amino acid concentrations with the odds of severe fatigue.

	Model 1		Model 2	
	Odds Ratio (95% Confidence Interval)	*p*-Value	Odds Ratio (95% Confidence Interval)	*p*-Value
**Essential**				
Histidine	1.21 (0.66; 2.38)	0.5	1.97 (0.91; 4.97)	0.1
Isoleucine	0.83 (0.42; 1.53)	0.6	0.88 (0.43; 1.71)	0.7
Leucine	0.75 (0.38; 1.37)	0.4	0.77 (0.37; 1.50)	0.5
Lysine	0.80 (0.41; 1.46)	0.5	0.78 (0.36; 1.51)	0.5
Methionine	0.80 (0.41; 1.47)	0.5	0.98 (0.47; 2.01)	0.9
Phenylalanine	0.95 (0.46; 1.85)	0.9	1.05 (0.48; 2.20)	0.9
Threonine	0.88 (0.48; 1.61)	0.7	1.05 (0.53; 2.15)	0.9
Tryptophan	1.52 (0.81; 3.00)	0.2	1.70 (0.87; 3.66)	0.1
Valine	0.77 (0.41; 1.41)	0.4	0.79 (0.39; 1.53)	0.5
**Non-essential**				
Alanine	1.42 (0.80; 2.61)	0.2	1.44 (0.80; 2.73)	0.2
Arginine	0.63 (0.30; 1.21)	0.2	0.66 (0.28; 1.36)	0.3
Asparagine	0.70 (0.35; 1.29)	0.3	0.85 (0.38; 1.78)	0.7
Citrulline	0.92 (0.50; 1.66)	0.8	1.14 (0.58; 2.30)	0.7
Glutamic acid	1.03 (0.50; 1.84)	0.9	0.89 (0.40; 1.66)	0.7
Glutamine	0.99 (0.54; 1.80)	0.9	1.36 (0.67; 2.84)	0.4
Glycine	0.76 (0.39; 1.39)	0.4	0.92 (0.43; 1.87)	0.8
Ornithine	0.84 (0.44; 1.53)	0.6	0.91 (0.46; 1.74)	0.8
Proline	2.55 (1.17; 7.12)	**0.042**	2.97 (1.27; 9.26)	**0.032**
Serine	0.60 (0.30; 1.11)	0.1	0.71 (0.33; 1.39)	0.3
Taurine *	0.41 (0.17; 0.84)	**0.027**	0.30 (0.10; 0.70)	**0.014**
Tyrosine	0.98 (0.51; 1.86)	0.9	1.18 (0.57; 2.50)	0.7
**Average amino acids**				
BCAAs	0.77 (0.40; 1.41)	0.4	0.80 (0.39; 1.54)	0.5
Essential	0.83 (0.44; 1.51)	0.6	0.92 (0.47; 1.75)	0.8
Non-essential	1.05 (0.57; 1.89)	0.5	1.15 (0.63; 2.16)	0.6
Total amino acids	0.99 (0.54; 1.78)	0.9	1.10 (0.60; 2.08)	0.7

* Log_2_-transformed prior to analyses. Model 1: Adjusted for age, sex, body mass index, and dialysis vintage. Model 2: Adjusted for age, sex, body mass index, dialysis vintage, hemoglobin concentration, C-reactive protein concentration, presence of cardiovascular disease, and presence of diabetes. Abbreviations: BCAAs: Branched chain amino acids. Statistically significant *p*-values (<0.05) are indicated in bold.

**Table 6:** Logistic regression analyses of daily amino acid losses and protein intake with severe fatigue.

	Model 1		Model 2	
Daily Amino Acid Losses	OR (95% CI)	*p*-Value	OR (95% CI)	*p*-Value
**Essential**				
Histidine	0.93 (0.47; 1.81)	0.8	0.95 (0.46; 1.88)	0.9
Isoleucine	0.75 (0.38; 1.37)	0.4	0.62 (0.28; 1.21)	0.2
Leucine	0.68 (0.34; 1.27)	0.2	0.54 (0.24; 1.09)	0.1
Lysine	0.79 (0.40; 1.49)	0.5	0.60 (0.26; 1.24)	0.2
Methionine	0.70 (0.35; 1.31)	0.3	0.71 (0.34; 1.36)	0.3
Phenylalanine	0.90 (0.45; 1.64)	0.7	0.80 (0.37; 1.51)	0.5
Threonine	0.88 (0.45; 1.68)	0.7	0.82 (0.40; 1.64)	0.6
Tryptophan *	1.31 (0.40; 5.00)	0.6	1.18 (0.35; 4.55)	0.8
Valine	0.74 (0.38; 1.38)	0.4	0.60 (0.27; 1.19)	0.2
**Non-essential**				
Alanine	1.26 (0.68; 2.37)	0.5	1.20 (0.61; 2.38)	0.6
Arginine	0.86 (0.46; 1.62)	0.6	0.82 (0.40; 1.64)	0.6
Asparagine	0.74 (0.36; 1.47)	0.4	0.77 (0.34; 1.61)	0.5
Citrulline	0.84 (0.46; 1.50)	0.6	0.81 (0.44; 1.46)	0.5
Glutamic acid	0.84 (0.44; 1.53)	0.6	0.41 (0.15; 0.97)	0.06
Glutamine	0.99 (0.52; 1.89)	0.9	1.01 (0.51; 1.98)	0.9
Glycine	0.67 (0.33; 1.28)	0.2	0.62 (0.27; 1.34)	0.2
Ornithine	0.79 (0.40; 1.50)	0.5	0.71 (0.33; 1.40)	0.3
Proline	1.46 (0.78; 2.83)	0.2	1.50 (0.77; 3.04)	0.2
Serine	0.89 (0.47; 1.63)	0.7	0.97 (0.49; 1.85)	0.9
Taurine *	0.69 (0.47; 0.97)	**0.043**	0.64 (0.42; 0.93)	**0.027**
Tyrosine	0.83 (0.44; 1.47)	0.5	0.78 (0.42; 1.41)	0.4
**Average amino acids**				
BCAAs	0.72 (0.36; 1.33)	0.3	0.58 (0.26; 1.15)	0.1
Essential	0.77 (0.39; 1.44)	0.4	0.65 (0.30; 1.28)	0.2
Non-essential	0.98 (0.51; 1.84)	0.9	0.94 (0.47; 1.86)	0.9
Total amino acids	0.91 (0.47; 1.70)	0.8	0.84 (0.42; 1.64)	0.6
**Dietary intake**				
Protein intake	0.18 (0.04; 0.50)	**0.003**	0.18 (0.04; 0.54)	**0.007**
Protein intake per kg bodyweight	0.22 (0.07; 0.53)	**0.003**	0.23 (0.07; 0.57)	**0.006**

* Log_2_-transformed prior to analyses. Model 1: Adjusted for age, sex, body mass index, and dialysis vintage. Model 2: Adjusted for age, sex, body mass index, dialysis vintage, hemoglobin concentration, C-reactive protein concentration, presence of cardiovascular disease, and presence of diabetes. Abbreviations: BCAAs: Branched chain amino acids. Statistically significant *p*-values (<0.05) are indicated in bold.

## Data Availability

Data described in the manuscript, code book, and analytic code will be made available upon request of the editor.

## Supplementary Materials

The following supporting information can be downloaded at: https://www.mdpi.com/article/10.3390/nu14142810/s1: Table S1. Daily amino acid losses (in grams and as a percentage of daily protein intake) in hemodialysis patients and controls. Table S2. Linear regression analyses on the determinants of the daily amino acid losses in hemodialysis patients. Table S3. Single dialysis losses, dialytic clearance, and fractional clearances of amino acids in hemodialysis patients. Table S4. Daily dialysis losses and urinary losses of amino acids in hemodialysis patients with residual diuresis. Figure S1. Graphical representation of the association of protein intake (A) and protein intake indexed to bodyweight (B) with the probability of severe fatigue. The analyses are adjusted for age, sex, body mass index, dialysis vintage, hemoglobin concentration, C-reactive protein concentration, presence of cardiovascular disease, and presence of diabetes. Table S5. Sensitivity analyses of the logistic regression analyses with severe fatigue.
