# Supplementary material for: Amino Acid Homeostasis and Fatigue in Chronic Hemodialysis Patients
Source: Nutrients. 2022 Jul 8;14(14):2810. doi: 10.3390/nu14142810 (PMC9318329; doi:10.3390/nu14142810)
Supplement: Supplementary file 1 [file nutrients-14-02810-s001.zip › nutrients-1786245-supplementary.pdf]

Table S1. Daily amino acid losses (in grams and as a percentage of daily protein intake) in hemodialysis patients and controls.

**Absolute amino acid losses**	**Hemodialysis patients**	**Controls**	**Ratio of hemodialysis to controls**	**P-value**
BCAA	0.54 ± 0.21	0.02 ± 0.01	34	<0.001
Total essential	1.24 ± 0.45	0.22 ± 0.13	6	<0.001
Total nonessential	2.77 ± 0.88	0.37 ± 0.19	7	<0.001
Total all amino acids	4.01 ± 1.29	0.60 ± 0.30	7	<0.001
**Fractional amino acid losses (% of protein intake)**				
BCAA	0.9 ± 0.3	0.02 ± 0.01	45	<0.001
Total essential	2.0 ± 0.7	0.3 ± 0.1	7	<0.001
Total nonessential	4.6 ± 1.8	0.4 ± 0.2	12	<0.001
Total all amino acids	6.7 ± 2.4	0.7 ± 0.3	10	<0.001

Abbreviations: BCAA: branched chain amino acids.

Table S2. Linear regression analyses on the determinants of the daily amino acid losses in hemodialysis patients.

**Determinants**	**Univariable**		**Multivariable regression**	
	**Std. β**	**P-value**	**Std. β**	**P-value**
Age	-0.27	**0.04**	0.01	0.9
Sex *	-0.46	0.09		
Body mass index	0.15	0.3		
Kt/V	0.46	**0.001**	0.51	**<0.001**
Dialysis vintage **	0.05	0.5		
Residual diuresis *	-0.42	0.1		
Plasma Hs-CRP **	-0.04	0.6		
Plasma albumin	0.31	**0.02**	0.01	0.9
Creatinine excretion	0.35	**0.006**	0.28	0.2
Protein intake	0.32	**0.02**	0.21	0.3

* Standardized beta does not represent scaled data but the beta for female sex and the presence of residual diuresis. ** Data presented for log_2-transformed data. Abbreviations: CRP: C-reactive protein.

Table S3. Single dialysis losses, dialytic clearance and fractional clearances of amino acids in hemodialysis patients.

Metabolite	Single dialysis losses (µmol)	Dialytic clearance (ml/min)	Fractional clearance (%)
Creatinine	15450 ± 8350	137 ± 68	100%
**Essential**			
Histidine	1940 ± 624	133 ± 32	103 ± 22
Isoleucine	1890 ± 786	116 ± 40	90 ± 27
Leucine	3265 ± 1376	121 ± 40	94 ± 27
Lysine	3717 ± 1384	114 ± 32	89 ± 21
Methionine	400 ± 221	88 ± 42	67 ± 28
Phenylalanine	1911 ± 797	120 ± 35	93 ± 22
Threonine	2445 ± 1050	121 ± 35	94 ± 22
Tryptophan	497 ± 142	80 ± 21	63 ± 17
Valine	5011 ± 1897	126 ± 35	98 ± 22
**Non-essential**			
Alanine	9058 ± 3532	130 ± 40	100 ± 25
Arginine	1939 ± 871	119 ± 41	93 ± 31
Asparagine	1723 ± 664	141 ± 44	110 ± 26
Citrulline	995 [769; 1310]	71 [54; 113]	56 [40; 88]
Glutamic acid	2262 ± 1302	81 ± 35	62 ± 27
Glutamine	16288 ± 5423	142 ± 38	111 ± 22
Glycine	7541 ± 2940	138 ± 27	106 ± 27
Ornithine	1268 ± 453	94 ± 27	72 ± 18
Proline	8325 ± 3014	133 ± 36	103 ± 23
Serine	1842 ± 850	129 ± 51	101 ± 46
Taurine	307 [56; 549]	20 [8; 51]	17 [6; 37]
Tyrosine	1067 ± 452	101 ± 37	78 ± 25
**Average amino acids**			
BCAA	10167 ± 3983 (= 1.26 ± 0.49 grams)	121 ± 38	94 ± 25
Essential	21076 ± 7548 (= 2.88 ± 1.02 grams)	113 ± 33	88 ± 21
Nonessential	52829 ± 16642 (= 6.40 ± 2.01 grams)	110 ± 33	85 ± 21
All amino acids	73905 ± 23295 (= 9.28 ± 2.93 grams)	112 ± 32	86 ± 21

Data were presented as mean ± standard deviation for data with a normal distribution and as median [interquartile range] for data not normally distributed. Abbreviations: BCAA: branched chain amino acids.

Table S4. Daily dialysis losses and urinary losses of amino acids in hemodialysis patients with residual diuresis.

Metabolite	Daily dialysis losses (µmol/24 h)	Daily urinary losses (µmol/24 h)	Ratio urinary dialysis losses to urinary losses
**Essential**			
Histidine	763 ± 255	81 ± 62	10 [6; 26]
Isoleucine	744 ± 346	4 [2; 11]	158 [68; 422]
Leucine	1278 ± 589	9 [3; 27]	106 [47; 427]
Lysine	1463 ± 584	75 [38; 151]	18 [9; 37]
Methionine	153 ± 91	5 [3; 9]	29 [14; 39]
Phenylalanine	775 ± 395	14 [5; 35]	46 [24; 114]
Threonine	932 ± 471	33 [6; 92]	23 [9; 140]
Tryptophan	200 ± 70	7 [2; 21]	27 [10; 125]
Valine	1995 ± 851	10 [4; 35]	145 [67; 381]
**Non-essential**			
Alanine	3562 ± 1453	131 [69; 217]	26 [15; 63]
Arginine	745 ± 344	8 [3; 17]	68 [39; 224]
Asparagine	629 ± 241	18 [4; 53]	27 [13; 190]
Citrulline	423 ± 219	18 [4; 41]	24 [9; 83]
Glutamic acid	912 ± 588	17 [8; 27]	57 [20; 113]
Glutamine	6435 ± 2602	54 [12; 179]	107 [34; 535]
Glycine	2958 ± 1344	558 [279; 857]	5 [3; 11]
Ornithine	521 ± 179	8 [3; 27]	55 [24; 131]
Proline	3317 ± 1271	114 [22; 206]	26 [15; 144]
Serine	675 ± 295	41 [11; 94]	15 [7; 81]
Taurine	123 [16; 246]	12 [7; 26]	6 [2; 19]
Tyrosine	426 ± 214	18 [8; 31]	17 [10; 44]
**Average amino acids**			
BCAA	4018 ± 1761	24 [9; 74]	132 [59; 363]
Essential	8303 ± 3398	232 [104; 531]	27 [16; 71]
Nonessential	20751 ± 7319	1235 [532; 1882]	16 [10; 43]
All amino acids	29054 ± 10351	1562 [670; 2376]	17 [12; 48]

Data were presented as mean ± standard deviation for data with a normal distribution and as median [interquartile range] for data not normally distributed. Abbreviations: BCAA: branched chain amino acids.

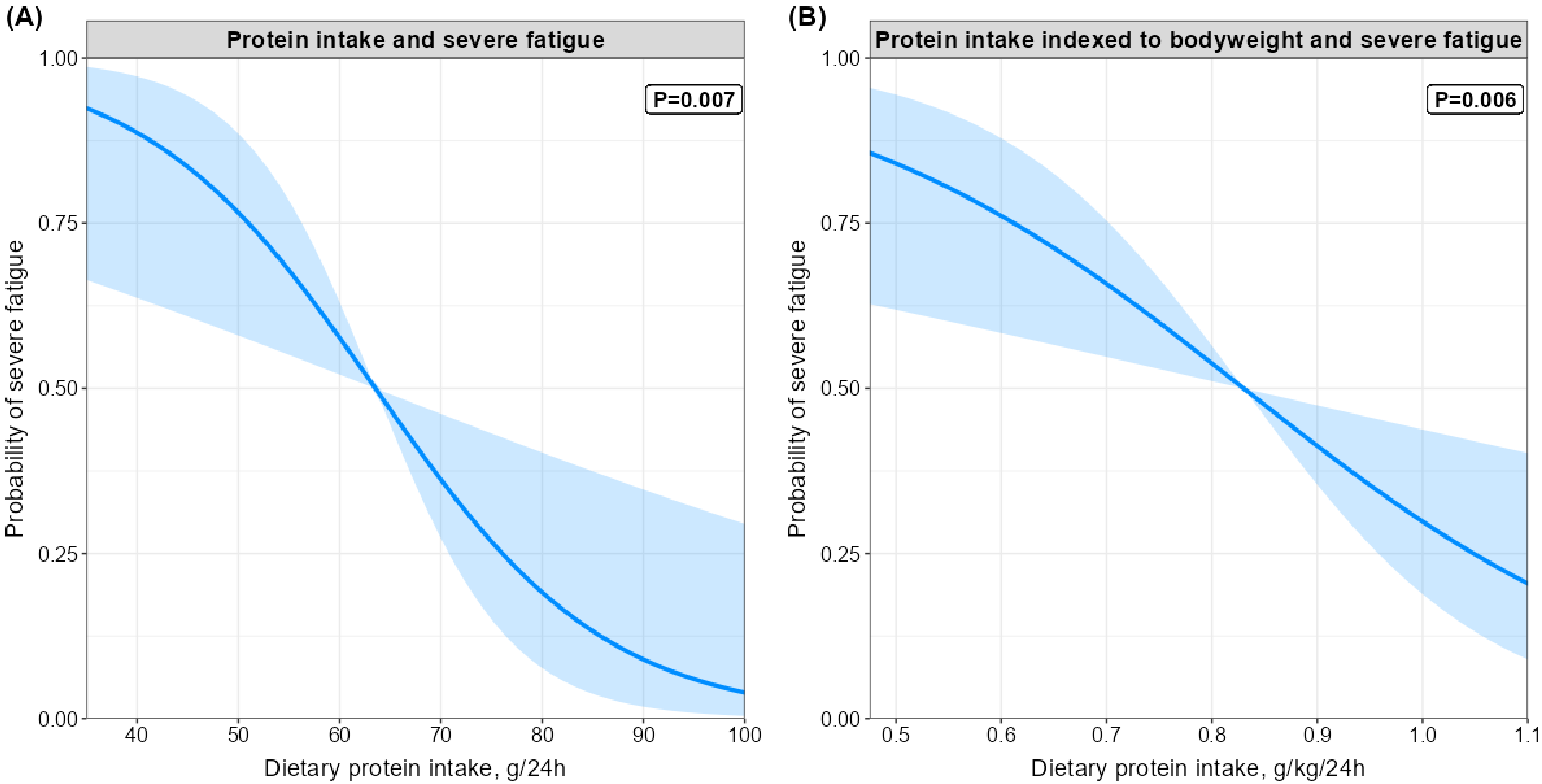

Figure S1. Graphical representation of the association of protein intake (A) and protein intake indexed to body weight (B) with the probability of severe fatigue. The analyses are adjusted for age, sex, body mass index, dialysis vintage, hemoglobin concentration, C-reactive protein concentration, presence of cardiovascular disease, and presence of diabetes.

Table S5. Sensitivity analyses of the logistic regression analyses with severe fatigue.

	Excluding outliers in the concerned variable *		Excluding patients with 2 dialysis sessions per week		Excluding patients with >60 months dialysis vintage		Excluding participants with BMI < 18.5		Excluding participants with low hemoglobin **	
	OR [95% CI]	P-value	OR [95% CI]	P-value	OR [95% CI]	P-value	OR [95% CI]	P-value	OR [95% CI]	P-value
Plasma taurine concentration	0.35 [0.10 ; 0.85]	0.045	0.27 [0.08 ; 0.66]	0.011	0.29 [0.08 ; 0.75]	0.027	0.30 [0.10 ; 0.71]	0.015	0.30 [0.09; 0.74]	0.023
Plasma proline concentration	2.03 [0.96 ; 4.91]	0.082	2.69 [1.15 ; 8.45]	0.053	2.15 [0.98 ; 6.44]	0.10	2.94 [1.25 ; 9.31]	0.035	3.03 [1.26; 10.1]	0.038
Daily taurine losses	0.68 [0.43 ; 1.02]	0.069	0.66 [0.43 ; 0.95]	0.033	0.42 [0.21 ; 0.72]	0.005	0.65 [0.42 ; 0.93]	0.029	0.68 [0.44; 0.99]	0.050
Protein intake	0.28 [0.09 ; 0.68]	0.010	0.18 [0.04 ; 0.56]	<0.001	0.08 [0.01 ; 0.35]	0.005	0.18 [0.04 ; 0.55]	0.008	0.19 [0.04; 0.60]	0.013
Protein intake per kg bodyweight	0.25 [0.08 ; 0.60]	0.006	0.23 [0.06 ; 0.59]	0.008	0.09 [0.01 ; 0.33]	0.003	0.21 [0.06 ; 0.57]	0.006	0.23 [0.07; 0.63]	0.012

* Defined as all values deviating more than two standard deviations from the mean. Analyses are adjusted for age, sex, body mass index, dialysis vintage, hemoglobin concentration, C-reactive protein concentration, presence of cardiovascular disease, and presence of diabetes. Abbreviations: BMI: Body mass index.

** Defined as the lowest 5th percentiles in males and females.
